# Correction: Comparison of six methods for *Loa loa* genomic DNA extraction

**DOI:** 10.1371/journal.pone.0291986

**Published:** 2023-09-19

**Authors:** The PLOS ONE Editors

## Notice of Republication

This article was republished on September 12^th^, 2023, to add Elsa-Rush Eyang-Assengone, Patrice Makouloutou-Nzassi, and Félicien Bangueboussa as the second, third, and fourth authors at the recommendation of a representative of Centre International de Recherches Médicales de Franceville. The About the Authors section and citation details were updated accordingly. Please download this article again to view the correct version. The originally published, uncorrected article and the republished, corrected article are provided here for reference.

RD and JPA stand by the original author list.

## Supporting information

S1 File Originally published, uncorrected article. (PDF)

S2 File Republished, corrected article. (PDF)
