# Supplementary material for: Comparison of six methods for Loa loa genomic DNA extraction
Source: PLoS One. 2022 Mar 21;17(3):e0265582. doi: 10.1371/journal.pone.0265582 (PMC8936488; doi:10.1371/journal.pone.0265582)
Supplement: S1 Table — (DOCX) [file pone.0265582.s001.docx]

**Table S1:formula for calculation of ratio, yield and interpretation**

**Calculation of DNA absorbance ratios:**

Ratio 260/280= (A_260_-A_320_)/(A_280_-A_320_)

Ratio 260/230= (A_260_-A_320_)/(A_230_-A_320_)

**Interpretation of values of ratio:**

|  |  |  |  |  |  |  |  |  |
| --- | --- | --- | --- | --- | --- | --- | --- | --- |
| **Ratio** | **Value** | **Indication of purity** | | | | | | |
| 260/280 | 1.8 | Pure DNA | | | | | | |
|  | < 1.8 | Presence of proteins, phenol, and other contaminants | | | | | | |
|  | > 1.8 | RNA contamination | | | | | | |
| 260/230 | 1.8–2.2 | Pure DNA | | | | | | |
|  | < 1.8 | Co-purified contaminants (solvents, salts, organic contaminants) | | | | | | |
|  |  |  |  |  |  |  |  |  |

**Calculation of DNA yield by technique:**

Yield (µg)= Concentration DNA (µg/ml) x Total volume of sample (ml)
