# Supplementary material for: Comparison of six methods for Loa loa genomic DNA extraction
Source: PLoS One. 2022 Mar 21;17(3):e0265582. doi: 10.1371/journal.pone.0265582 (PMC8936488; doi:10.1371/journal.pone.0265582)
Supplement: S2 Table — (DOCX) [file pone.0265582.s002.docx]

**Table S2 : Raw replicat data (OD) and concentration of extracted DNA**

|  |  |  |  |  |  |  |  |  |  |  |  |  |
| --- | --- | --- | --- | --- | --- | --- | --- | --- | --- | --- | --- | --- |
|  |  | **O.D (NanoVue)** | | | | | | | | | | |
| **Method** | **Test** | **A 230** | **Ⴟ(O.D)** |  | **A 260** | **Ⴟ(O.D)** |  | **A 280** | **Ⴟ(O.D)** |  | **A 320** | **Ⴟ(O.D)** |
| Phenol/Chloroform | Test 1 | 16.66 | **17.835** |  | 16.55 | **18.77** |  | 10.4 | **14.58** |  | 4.23 | **9.34** |
|  | Test 2 | 19.01 |  |  | 20.99 |  |  | 18.76 |  |  | 14.45 |  |
| Qiagen | Test 1 | 7.41 | **6.561** |  | 9.55 | **8.125** |  | 6.39 | **5.265** |  | 2.94 | **2.205** |
|  | Test 2 | 5.712 |  |  | 6.7 |  |  | 4.14 |  |  | 1.47 |  |
| Salting-out | Test 1 | 4.37 | **4.17** |  | 7.74 | **7.415** |  | 4 | **4.4** |  | 0.64 | **1.05** |
|  | Test 2 | 3.97 |  |  | 7.09 |  |  | 4.8 |  |  | 1.46 |  |
| Tris-EDTA | Test 1 | 26.24 | **26.115** |  | 29.33 | **29.2** |  | 20.58 | **19.26** |  | 7.32 | **9.26** |
|  | Test 2 | 25.99 |  |  | 29.07 |  |  | 17.94 |  |  | 11.2 |  |
| Methanol | Test 1 | 7.48 | **6.299** |  | 9.1 | **7.928** |  | 4.97 | **4.55** |  | 0.55 | **1.55** |
|  | Test 2 | 5.118 |  |  | 6.756 |  |  | 4.13 |  |  | 2.55 |  |
| CTAB | Test 1 | 6.09 | **5.725** |  | 14.39 | **13.405** |  | 7.16 | **6.84** |  | 0.57 | **0.34** |
|  | Test 2 | 5.36 |  |  | 12.42 |  |  | 6.52 |  |  | 0.11 |  |
|  |  |  |  |  |  |  |  |  |  |  |  |  |

|  |  |  |  |
| --- | --- | --- | --- |
|  |  | **Quantity (Qubit® 2.0)** | |
| **Method** | **Test** | **C° (ng/µl)** | **Ⴟ(C°µg)** |
| Phenol/Chloroform | Test 1 | 35.9 | **39.2** |
|  | Test 2 | 42.5 |  |
| Qiagen | Test 1 | 52 | **51.4** |
|  | Test 2 | 50.8 |  |
| Salting-Out | Test 1 | 51 | **51.95** |
|  | Test 2 | 52.9 |  |
| Tris-EDTA | Test 1 | 8.69 | **8.505** |
|  | Test 2 | 8.32 |  |
| Methanol | Test 1 | 1.19 | **1.595** |
|  | Test 2 | 2 |  |
| CTAB | Test 1 | 11.4 | **11.15** |
|  | Test 2 | 10.9 |  |
|  |  |  |  |
