# Supplementary material for: Comparison of six methods for Loa loa genomic DNA extraction
Source: PLoS One. 2022 Mar 21;17(3):e0265582. doi: 10.1371/journal.pone.0265582 (PMC8936488; doi:10.1371/journal.pone.0265582)
Supplement: S1 Raw images — Fig 1. Comparison of six methods of Loa loa DNA extraction. PM = standard molecular weight. Lines 1–2: DNA from phenol/chloroform; 3–4: Qiagen extract; 5–6: salting-out extract; 7–8: Tris-EDTA extract; 9–10: methanol extract; 11–12: CTAB extract. The values on the left are the size of molecules. Fig 2. Digestion of extracted Loa loa DNA by EcoRI and BamHI endonuclease. 2A: Undigested (lines: 1-3-5-7-9-11) compared to digested with EcoRI (lines: 2-4-6-8-10-12) DNA; PM = molecular marker. 2B: Undigested (lines:1-3-5-7-9-11) compared to digested with BamHI (lines:2-4-6-8-10-12) DNA; extracts were analyzed via agarose gel electrophoresis (1%) and visualized under UV light. The values on the left indicate the size of the bands. PM = molecular marker. Fig 3. Amplification of DNA extracted by PCR. The methods are listed on the top of each band: phenol:chlorof line 1; Qiagen line 2; salting out line 3; Tris-EDTA line 4; Methanol line 5; CTAB line 6; band 7 is a negative control. PM = molecular marker. The values on the left represent the size of DNA. (PDF) [file pone.0265582.s003.pdf]

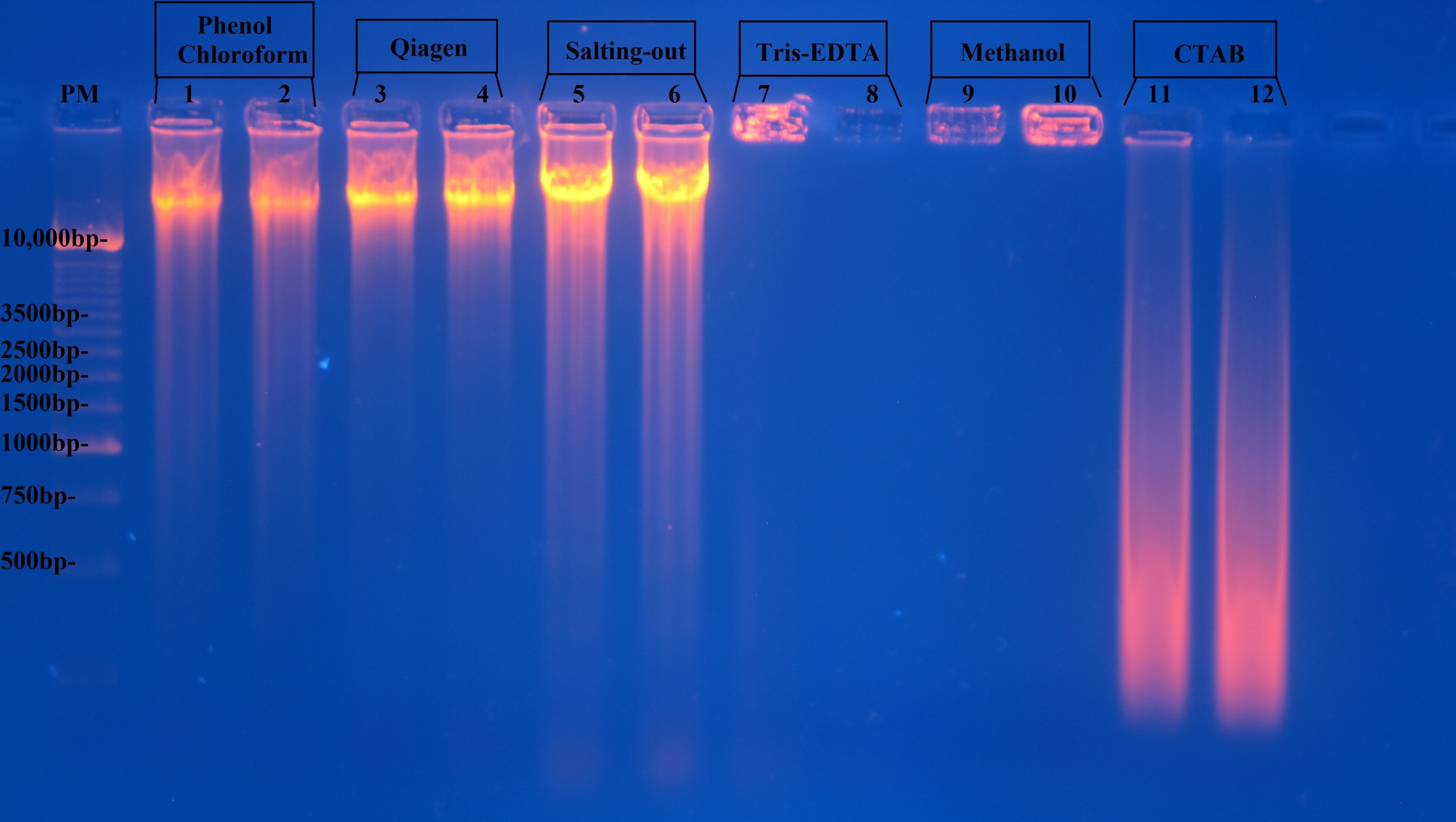

Fig 1. Comparison of six methods of *Loa loa* DNA

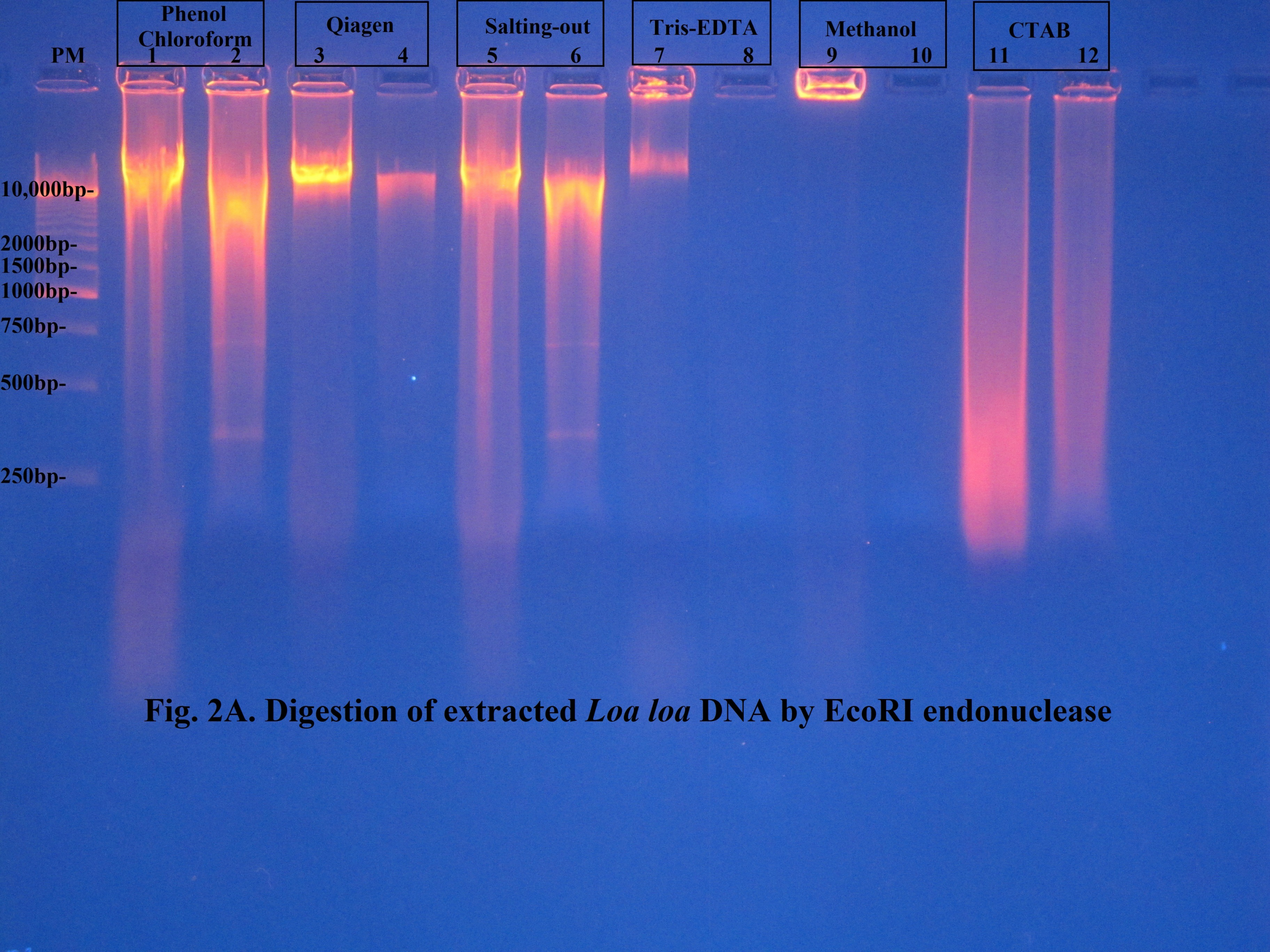

Fig. 2A. Digestion of extracted *Loa loa* DNA by EcoRI endonuclease

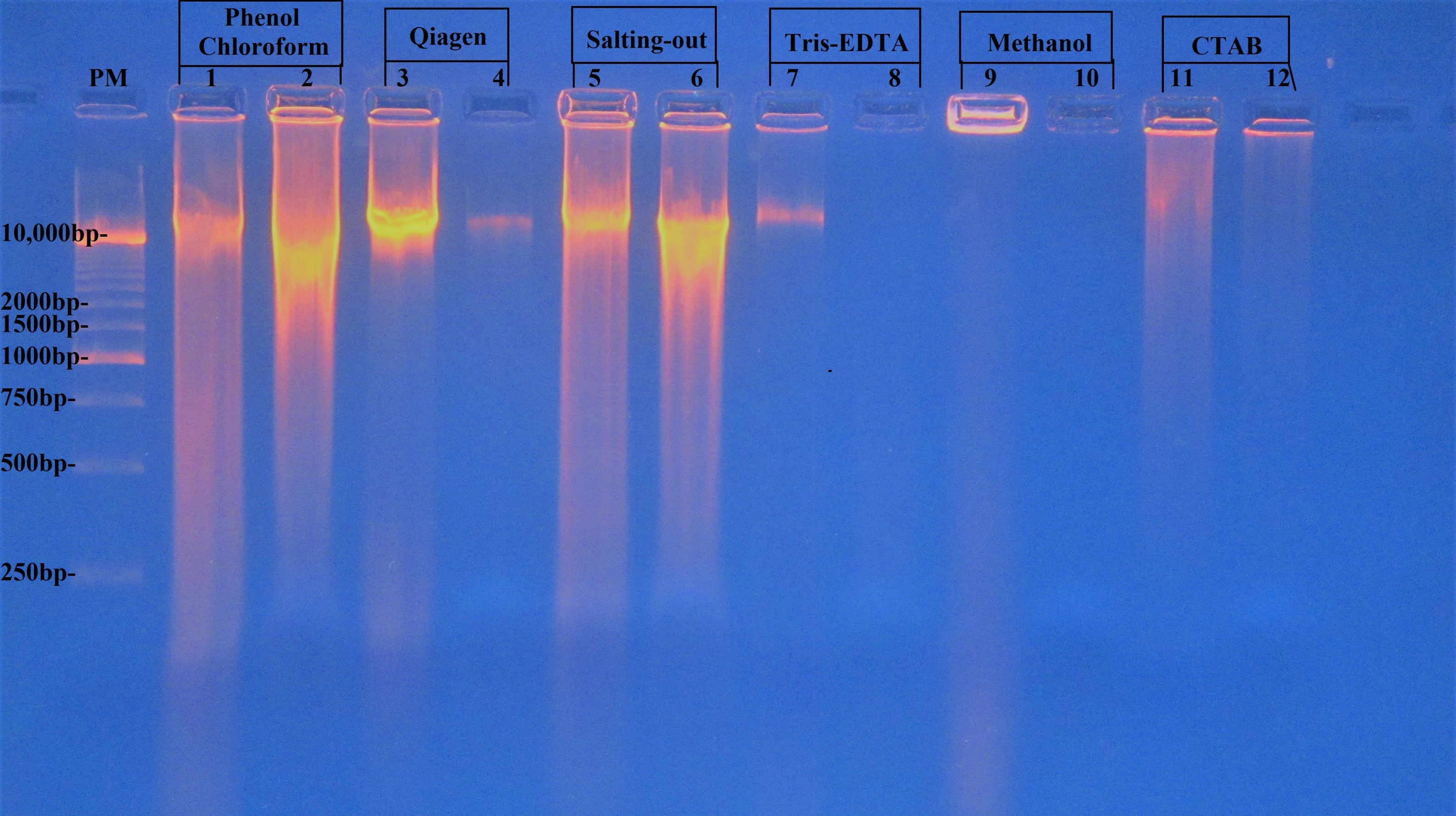

Fig 2B. Digestion of *Loa loa* DNA extracted by BamHI endonuclease

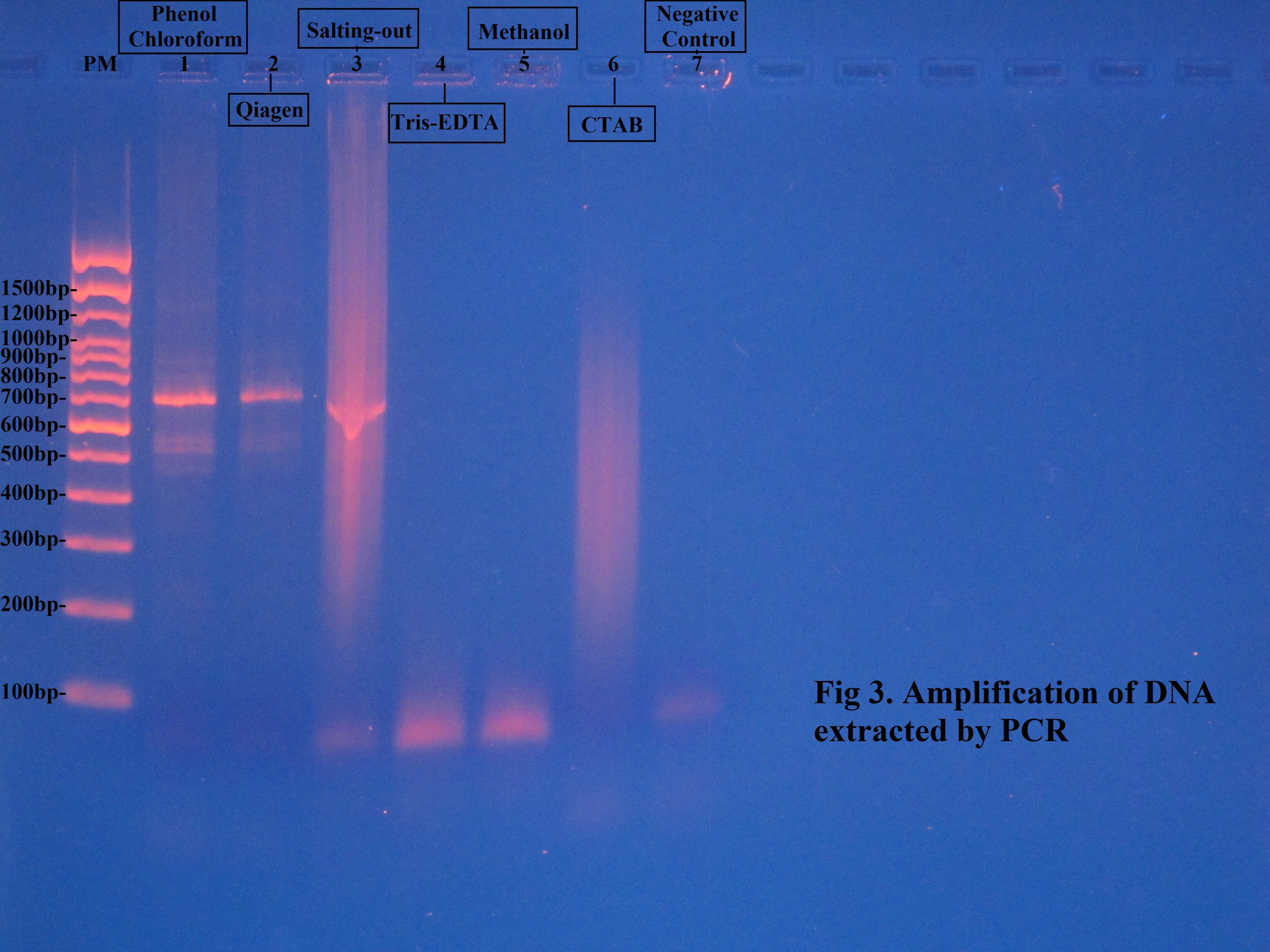

Fig 3. Amplification of DNA extracted by PCR
