# Supplementary material for: Comparison of six methods for Loa loa genomic DNA extraction
Source: PLoS One. 2022 Mar 21;17(3):e0265582. doi: 10.1371/journal.pone.0265582 (PMC8936488; doi:10.1371/journal.pone.0265582)
Supplement: Attachment — Submitted filename: Response to reviewer and editor, Plos One (DIEKI and JPA).docx [file pone.0265582.s004.docx]

**Response to Reviewers**

**A) Academic editor:**

1. The manuscript meets the requirements of PLOS One as indicated in the template.

2. We have addressed the information on funding. We did not have a grant for this work but internal funding and we have changed the information accordingly.

3. The ethics statement is now included in the Methods section (see Methods, page 5, lines 104–105).

4. The images provided in this article are original, uncropped, and unadjusted. We added supporting information to clarify the analysis: (1) one part of the supporting information (**S1**) is related to data on the calculation and interpretation of the optical density (OD) ratio and yield; (2) the second part is related to the raw OD (**S2**) data from all the experiments and the mean of the replicat (X) from each of the six methods, including the mean concentration for each extraction method.

5. The references have been completed by adding missing references in the original article (see page 5, line 109; page 7 line 131; page 8 line 160; page 11, line 233; page 11 line 234) and in reference section page 21, line 456-457; page 21, line 461-463, Line 464-466.

The PLOS data policy: The rest of the complementary data underlying the findings described in the manuscript are now available in the supporting information Table **S1** and Table **S2.**

B**. Reviewer 1**:

**Abstract:**

An abstract has been included in the main manuscript (pages 2 and 3, line 23–56)

DNA extraction: The term “vaccine delivery” has been replaced by “**development**” (see line 25, page 2).

**Results**: The presentation of the results has been shortened to avoid repetition (see page 2, line 40–42).

**Introduction:**

***Loa loa***: The term has been written out, instead of ***L. loa****,* the first time it is used (see page 4, line 65).

Line 50: The sentence has been changed in accordance with the reviewer’s comments (see line 91, page 5).

**Methods:**

Human participants: A sentence has been added to address this point (see page 5, line 104–109),

Line 77: Since the meaning of the sentence seemed to be ambiguous, it has been rewritten (see page 6, line 124–125).

Line 84: The reference has been added (see page 7, line 131) and included in the reference section (see page 21, line 456).

Line 86: This has been corrected (see page 7, line 133–134).

Line 92: This is now specified (see page 7, line 139-140).

Line 95: The protocol is now mentioned (see page 7, line 143-144).

Line 99: There is now a space between temperature and “for” (see page 7, line 148).

Line 100: There is now a space between numbers and units (see page 7, line 148).

Line 107: The storage conditions are now specified (see page 8, line 156).

Line 112: The reference was added in the text (see page 8, line 160) and in the reference section; page 21, line 457-458.

Line 166: Because of confusion in the original form, we have changed the presentation and switched the PCR and electrophoresis section around as recommended by the reviewer (page 11, line 224–242).

Line 179/180: A space has now been inserted between all numbers and units (see page 11, line 227-228)

Line 184: The origin of primers, the reference paper, and the target gene are now specified (page 11, line 233-234).

Discussion:

Line 284: Identification of the contaminant was carried out using the classic parameters described in supporting information **Table S1** backed by raw data in supporting information Table S**2.** Page **15, line 319-321 and in section material and method page 10, line 214-215.**

Line 309: The activity is now specified in line 356-358, page 17

As suggested by the reviewer, we now refer to the figures and table in the relevant parts of the discussion section.

Line 311: To avoid any bias that may arise from the part on “band intensity by eye,” the sentence was removed from the original text (see page 19, line 403–405).

Line 314: The statement on “band distortion” has been clarified (page 17, line 362–366).

Line 323: Table 1 is referred to systematically throughout the **discussion section** wherever the concentration or yield is mentioned.

Line 338: This detail has been added (see page 15, line 307; page 18, line 390).

Line 352: The sentence has been modified according to the reviewer’s comments (see page 19, line 403–405).

-The Qiagen kit was chosen on the basis of availability and because this kit is typically used in our laboratory.

- As the reviewer may know, figures were not included in the text because this is a requirement of the journal. WE think that the reviewer and editor will understand why. Furthermore, we now refer to the figure and the table wherever possible.

**C. Reviewer 2:**

The reviewer notes several inconsistencies such as in the reporting of the 260/280 ratio. This has been corrected (page 12, line 247-248).

In line 197, “far behind those of” has been changed to “far greater than” (see page 12, line 254). Also, to address some other inconsistencies and redundancies, the whole section has been rewritten and is now entitled, as suggested by the reviewer, “Comparative spectrometric analysis of the six methods” (page 12, line 245–264.)
